# The Rhesus Macaque as a Translational Model for Neurodegeneration and Alzheimer’s Disease

**DOI:** 10.3389/fnagi.2021.734173

**Published:** 2021-09-03

**Authors:** Gail A. Stonebarger, Heather A. Bimonte-Nelson, Henryk F. Urbanski

**Affiliations:** ^1^Division of Neuroscience, Oregon National Primate Research Center, Beaverton, OR, United States; ^2^Department of Behavioral Neuroscience, Oregon Health and Science University, Portland, OR, United States; ^3^Department of Psychology, Arizona State University, Tempe, AZ, United States; ^4^Arizona Alzheimer’s Consortium, Phoenix, AZ, United States

**Keywords:** amyloid beta, animal models, brain aging, clinical aging, cognitive decline, non-human primate, phosphorylated tau

## Abstract

A major obstacle to progress in understanding the etiology of normative and pathological human brain aging is the availability of suitable animal models for experimentation. The present article will highlight our current knowledge regarding human brain aging and neurodegeneration, specifically in the context of Alzheimer’s disease (AD). Additionally, it will examine the use of the rhesus macaque monkey as a pragmatic translational animal model in which to study underlying causal mechanisms. Specifically, the discussion will focus on behavioral and protein-level brain changes that occur within the central nervous system (CNS) of aged monkeys, and compare them to the changes observed in humans during clinically normative aging and in AD.

## Introduction

### Alzheimer’s Disease

By the year 2050, nearly one in four people in the United States is expected to be 65 years of age or older, and people over 65 are predicted to make up 61.9% of the global population (U.S. Census Bureau, 2020). This change in population dynamics is likely to have a major impact on the health care systems. For instance, even in the absence of overt neurodegeneration, older adults show gradual changes in mental processes such as attention, memory retrieval, processing speed, and executive function (Harada et al., 2013; Schott, 2017). However, half of adults in the United States aged 50–64 years consider themselves at least somewhat at risk for developing overt dementia such as Alzheimer’s disease (AD), and 37% report having a family member with dementia (Maust et al., 2020). These concerns encompass the wide range of AD symptoms—including progressive memory loss, agitation, language deficits, depression, mood disturbances, and even psychosis—and are not unfounded, as a growing percentage of the population does develop AD (Schott, 2017; Mehla et al., 2020). The most common symptoms of AD are the severe dementia and neuropsychiatric complaints involved in advanced AD, with 60–70% of dementia cases being attributable to AD (Dey et al., 2017).

### Pathological Hallmarks of AD in Humans

**FIGURE 1:**
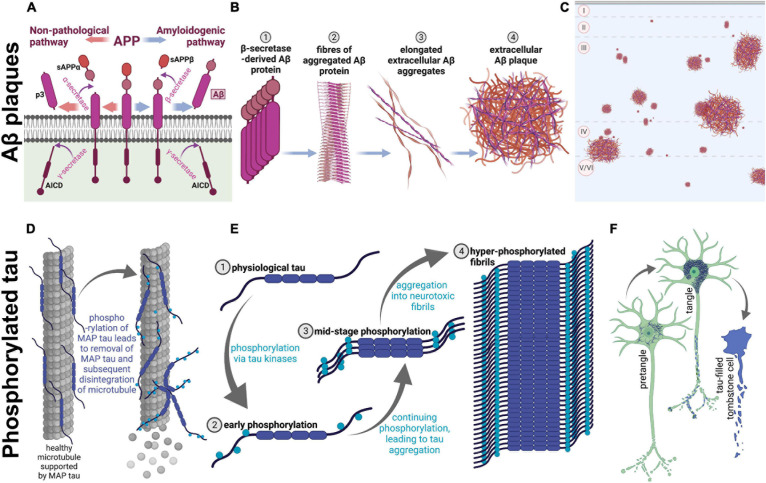
Amyloidogenesis and tau tangle formation across aging. Physiological proteins amyloid precursor protein (APP) and microtubule-associated protein tau undergo modifications during aging to form plaques and tangles, respectively. These processes occur in both humans and rhesus macaques across aging, resulting in parallel pathological aggregations of the same proteins in both species. **(A–C)** Show amyloidogenesis, while **(D–F)** represent processes involved in tau pathology. **(A)** Prior to aging, the transmembrane amyloid precursor protein is cleaved by α-secretase to form soluble amyloid precursor protein α (sAPPα; orange arrows; left), and subsequently cleaved further by γ-secretase, which creates p3 and APP intracellular domain (AICD). On the other hand, when APP is cleaved by β-secretase (blue arrows; right), soluble APPβ (sAPPβ) is formed before γ-secretase cleavage into AICD and Aβ, an insoluble pathological protein which is cleaved into the extracellular space. sAPPα, sAPPβ, and p3 are non-pathological APP fragments released extracellularly, while AICD is also physiologically relevant and is released into the intracellular matrix. **(B)** Aβ proteins begin to aggregate (1), create fibrils (2), form elongated fibers (3), and finally these fibers aggregate into insoluble extracellular plaques in the cortex (4). **(C)** A graphical representation of extracellular Aβ plaques in the neocortex. The top of the image represents the pial surface, with dashed horizontal lines signifying boundaries between cortical layers. Plaques vary in size and can be present in cortical layers II-VI. **(D)** On the left, microtubule-associated protein tau (tau) supports microtubules, a part of the neuronal cytoskeleton. When tau begins to be phosphorylated (on the right), the protein no longer adheres to the microtubule, leading to disintegration of the microtubule and subsequent degeneration of the neurites. **(E)** Tau (1) phosphorylation begins with early phosphorylation sites, which remove the protein from its role providing support to microtubules (2). These phosphorylated proteins are therefore present in the intracellular space, and free to be further phosphorylated by various tau kinases, which leads to intracellular aggregation (3). Eventually, hyperphosphorylation leads to formation of tau fibrils (4), which aggregate intracellularly. **(F)** Graphical representation of the clinical progression of intracellular tauopathy. In all three neurons, blue represents phosphorylated tau (p-tau). First, p-tau accumulates in the cell, visible via cytoplasmic immunoreactivity, and can be considered a pretangle. It can be presumed that early axonal degeneration is occurring, due to accumulation of detached tau in the cytoplasm. Then, when tauopathy progresses, the cytoplasm shows heavy, fibrillous tau aggregations, which exclude the nucleus and usually present as a teardrop-like shape as seen in the middle neuron. Additionally, at this stage, dystrophic neurites are visibly tau-filled (represented by blue axonal inclusions) and the neuron is presumed to be primarily non-functional. Finally, p-tau continues to fill the intracellular space until the neuron dies. At this stage, the nucleus is no longer distinguishable, and the cell membrane may be “leaking” tau into the extracellular space. The first two phenotypes are seen in very old monkeys, but progression to tombstone cells is uncommon. Figure created with BioRender.com.

Many physical changes in the brain have been identified across clinically normative aging, such as mild brain atrophy, gliosis, and accumulation of intracellular and extracellular proteins (Marner et al., 2003; Freeman et al., 2008; Schott, 2017; Furcila et al., 2018). These changes do appear to alter cognitive function, even in healthy aging humans (Furcila et al., 2018). However, patients with AD show these symptoms at a younger age, which sets AD patients onto a separate, pathological, brain aging trajectory. AD patients additionally show overt neuron loss, which is not present in normative aging (Freeman et al., 2008). Accordingly, they also reach higher levels of pathology than their non-AD peers (Duyckaerts et al., 2009). Two hallmarks of AD pathology are amyloid beta (Aβ): A protein which aggregates into extracellular plaques and is formed from cleavage of the APP, and phosphorylated tau (p-tau): A microtubule-associated protein which becomes hyperphosphorylated and accumulates intracellularly (Duyckaerts et al., 2009; see Figure 1 for protein aggregation of each). Notably, these proteins are present in the non-AD aging brain as well; however, specific protein modifications are necessary for the clinical diagnosis of AD (Furcila et al., 2018).

### Current Pharmacological Therapies

There has been very limited efficacy in preventing the onset of some new memory-related symptoms with memantine, an *N*-methyl-D-aspartate (NMDA) receptor antagonist, cholinesterase inhibiters such as donepezil, and a recently approved combination of the two classes (Miziak et al., 2021). However, there are no current interventions which treat the etiology of or even prevent the progression of AD, in spite of many studies and clinical trials (i.e., Farlow et al., 2012; Ostrowitzki et al., 2017; Cummings et al., 2018; Egan et al., 2019; Haass and Levin, 2019). Most recently, aducanumab, an antibody shown to reduce amyloid beta accumulation, was FDA-approved using a fast-tracked pathway (U.S. Food & Drug Administration, 2021). This approval, however, was based solely on the surrogate endpoint of plaque reduction, so the therapeutic potential of aducanumab is unknown especially in the context of symptomatic relief. This is because Aβ plaques do not reliably correlate with cognitive decline, and this and previous amyloid-lowering drugs have failed to consistently slow dementia onset (Nelson et al., 2012; Sevigny et al., 2016). Therefore, to make significant advances in our understanding of the pathways and neurodegenerative trajectories that underlie AD, and to develop safe and effective therapies, it is important to develop and characterize appropriate experimental animal models of AD. These preclinical models inform and drive novel investigations in the clinical realm, underscoring the importance of promoting cross-talk between preclinical and clinical science, as well as the critical nature of translational science for therapeutics.

### Preclinical Rodent Models of AD

Rodents are the most commonly used laboratory animals and have relatively short lifespans to allow for rapid experimental turnaround in aging studies. They do not, however, naturally develop measurable pathological features of AD such as Aβ plaques or p-tau tangles (LaFerla and Green, 2012). Several methods have been employed to mitigate this lack of overt pathology, generally via modifications to genes which are known risk factors for AD, such as amyloid precursor protein (APP), apolipoprotein E, and presenilin (Webster et al., 2014). Other models involve adeno-associated virus (AAV) vectors and floxed genes (i.e., temporally controlled and mediated genes), induction of pathology via direct or indirect addition of Aβ and/or p-tau, or sometimes direct insertion of clinical genes or proteins found in AD patients. For examples of these manipulations, see Alzforum (2021). These models allow great flexibility and temporal control over induction of AD characteristics which cannot be achieved with sporadically occurring clinical AD. Specific realms of memory can be probed on a basic level using rodent behavioral paradigms; for instance: set-shifting, spatial navigation, working memory, perseveration, and many other typically affected forms of cognition are measurable in rodents. These paradigms provide valuable insights into specific realms of memory as well as molecular mechanisms of aging. Conversely, some complex cognitive measures—such as the neurocognitive testing considered to be the clinical gold standard—are impractical or sometimes even impossible to use in characterizations of AD-like symptomology in rodents (Harada et al., 2013; Webster et al., 2014; Deb et al., 2017; Ingram and De Cabo, 2017; Colman, 2018). Therefore, collectively, when assessing clinical hallmarks of AD, the rodent model provides a basis for informing studies in higher mammals.

Mice and rats are short-lived, allowing for study of the aging process in a relatively contracted time frame, but this abbreviated lifespan also limits chronological aging. For instance, 2 years (the average lifespan of a laboratory mouse; Flurkey et al., 2007) simply may not be enough time for pathological proteins such as Aβ or p-tau to accumulate and have neurodegenerative effects. As seen in the interactive visualization by Alzforum (2021), many mouse models do recapitulate aspects of AD—for example, APP processing (Figures 1A–C) via genetic induction—and they have been crucial to our understanding of mechanisms of aging and AD. To gain a holistic understanding of the disease, a more robust, naturally-occurring model is optimal. This model must also be highly clinically translational in order to probe complex cognitive functioning, temporal dynamics, and non-cognitive neuropsychiatric symptoms. These shortcomings in rodent models may be complemented with the study of the rhesus macaque to model AD and aging. Taken together, the use of small laboratory rodents is paramount for studying cellular and molecular aspects of AD pathology, but a more complete clinically translational model is needed to recapitulate subtle behavioral symptoms and sporadic onset of both AD and normative aging mechanisms.

## The Rhesus Macaque Model of Normative and Pathological Brain Aging

The rhesus macaque (*Macaca mulatta*) shows close genetic homology to humans (Rhesus Macaque Genome Sequencing Analysis Consortium, Gibbs et al., 2007), has a greatly extended lifespan relative to rodents (Mattison et al., 2017; Stonebarger et al., 2020), is capable of performing complex and prefrontal cortex (PFC)-dependent cognitive tests similar to those used in the clinic (Rapp, 1989; Peters et al., 1996; Moore et al., 2006; Nagahara et al., 2010) and also shows similar life stages; i.e., a prolonged childhood, adolescence, adulthood, and cognitive and hormonal aging declines (Downs and Urbanski, 2006; Messaoudi et al., 2011; Sorwell et al., 2012; Mattison et al., 2017). For these reasons, the rhesus macaque may represent a more holistic and systems approach to studying mechanisms underlying human aging. Additionally, it has been commonly known for decades that rhesus macaques develop cortical and subcortical Aβ plaques—which represents the natural formation and aggregation of this protein—and there is evidence that this involves the same pathways as in human brain aging (Heilbroner and Kemper, 1990; Uno, 1993; Stonebarger et al., 2020).

### Cognitive Decline

Because rhesus macaques are capable of more complex learning and memory tasks and have longer lifespans compared to rodents, they represent a valuable translational animal model with which to study longitudinal changes in cognitive function. For instance, many behavioral batteries include neuropsychological tests that closely resemble those used to clinically assess cognitive decline; i.e., monkeys are capable of performing testing with the same complexity and nearly identical parameters as multiple tests used in the clinic. To this end, many researchers have observed a gradual but significant decline in cognitive ability which occurs over the course of macaque late adulthood (Lai et al., 1995; Herndon et al., 1997; Moore et al., 2003, 2006). For example, short-term memory, task-dependent memory, two-choice discriminations, abstraction, and set-shifting have all been shown to be impaired in old rhesus macaques compared to young, or even in some instances also compared to middle-aged monkeys (Bartus et al., 1978; Rapp and Amaral, 1989; Lai et al., 1995; Herndon et al., 1997; Voytko, 1999; Moore et al., 2003). Importantly, as in humans, this decline is progressive such that “early aged” monkeys (19–23 years), “middle-aged” monkeys (24–29 years), and “oldest-old” (30+ years) show different levels of cognitive performance. Each age group shows impairments compared to the group directly younger in delayed non-match to sample tasks and non-spatial delayed recognition span test (Herndon et al., 1997; Harada et al., 2013). Like humans, variability in rhesus cognitive scores also increases with age (Schott, 2017). However, even considering deficits seen in animals with the lowest cognitive scores, aging macaques do not show decline of cognitive function as rapidly or to such severe impairment as humans with AD (Hara et al., 2012). After decades of cognitive aging research in the rhesus macaque, the primary takeaway points seem to be that the rhesus macaque does not model overt dementia such as AD, but instead models the natural decline in various cognitive domains seen in healthy aging humans. It remains to be seen whether pathological tauopathy is correlated with cognitive decline in the rhesus macaque, as amyloid plaques are not consistently predictive of cognitive decline in monkeys (Emborg, 2017).

### Amyloid Beta

For decades, the progression and accumulation of Aβ extracellular plaques in the rhesus macaque has been well-documented. For example, in 1993 Uno reported amyloid plaques in 40–66% of rhesus macaques aged 20–30 years (Uno, 1993), and as detection methods have improved in the last three decades, it may be that an even higher percentage of aged animals show amyloidosis. However, at least in the PFC, amyloidosis levels do not reach the threshold for clinical AD. For instance, brains of AD patients tend to show 10–20% area coverage of plaques in the PFC (O’Brien and Wong, 2011; Smith et al., 2019). Furthermore, it was recently shown that even the oldest known rhesus macaques in the world do not experience amyloid levels naturally higher than 5–6% coverage, depending on the region of interest (Stonebarger et al., 2020). These PFC data could indicate that globally, Aβ simply does not accumulate enough in the rhesus brain to be overtly neurotoxic. Nevertheless, a majority of studies using various methodologies have confirmed an increase in rhesus cerebral amyloid across age, which parallels findings in the clinic (Heilbroner and Kemper, 1990; Uno, 1993; Stonebarger et al., 2020). Moreover, building on this observation, there have been successful attempts to induce—rather than replicate—a phenotype that more closely parallels the human AD brain environment in multiple species of macaques (Forny-Germano et al., 2014; Beckman et al., 2019). Altogether, these Aβ plaque data strongly suggest that the same aging processes occur in both human and rhesus macaques, but that they may be less developed in the rhesus brain.

### Phosphorylated Tau

Until recently, the rhesus macaque had been accepted as an incomplete model of AD, or potentially even dismissed fully as a model of natural clinical aging, due to the lack of neuronal p-tau detection. Indeed, for decades, rhesus macaques were considered not to develop p-tau naturally. This has presented a challenge in phylogenetically synthesizing the evolution of p-tau tangles—as chimpanzees, baboons, gorillas, and cynomolgus monkeys have all shown evidence of cortical tauopathy (Schultz et al., 2000; Oikawa et al., 2010; Perez et al., 2013; Edler et al., 2020), along with some smaller simians such as marmosets and lemurs (Giannakopoulos et al., 1997; Sharma et al., 2019). Rhesus monkeys, a key evolutionary link, have not historically shown immunoreactivity to p-tau (Peters et al., 1996; Zhang et al., 2019). However, in 2018 Paspalas et al. reported detection of p-tau in the rhesus macaque entorhinal cortex, which has led to a resurgence in interest surrounding non-human primate p-tau. In addition to this initial landmark study, laboratories are currently researching both natural p-tau accumulation and tauopathy induction models in the rhesus macaque (i.e., Beckman et al., 2021; Datta et al., 2021). There is still much work to be done characterizing natural tauopathy in the rhesus macaque, but it has generally been observed that most monkeys do not live long enough to develop pathological tauopathy. For instance, most overt tauopathy appears to occur mainly in macaques over the age of 30 years, and very few primate facilities have a cohort of these very old animals (Paspalas et al., 2018; Datta et al., 2021). Therefore, non-human primate induction models become highly relevant: if the same natural pathways that lead to tauopathy can be dysregulated or stimulated earlier in life, researchers can study the etiology and progression of the proteinopathy across many years, and monkeys would likely achieve more advanced states of tauopathy. This could mimic neurodegeneration or advanced stages of human brain aging, which can inform clinical progression of the disease; if researchers can induce tauopathy into a further progressed state in the macaque, the inverse processes are logical targets for early AD intervention. It should be emphasized that relatively few rhesus monkeys live beyond ∼25 years, which is prior to the age at which they begin to show significant hippocampal tauopathy (Paspalas et al., 2018; Datta et al., 2021); thus, the systematic study of p-tau induction will be critically important in further developing the macaque AD model.

Taken together, the recent p-tau data question the traditional dogma that non-human primates do not develop AD due to a lack of p-tau accumulation. The primary physical AD hallmarks: clinically relevant p-tau intra-neuronal tangles and Aβ plaques, have now been observed in monkeys; so why don’t they show a full cognitive AD phenotype?

### Neuronal Death

Mid- and late- stage clinical AD result in significant neuronal death in subcortical structures such as the hippocampus, and in the neocortex (Furcila et al., 2018). To fully capitulate an AD phenotype, the rhesus macaque would need to show significant cell loss in areas that are sensitive to aging. Thus far, there is only one report of neuronal loss in the rhesus macaque with loss indicated in PFC Brodmann Area 8a (Smith et al., 2004). However, data from Stonebarger et al. (2020) included a larger cohort of monkeys across more of the lifespan, and this expanded cohort did not show age-related neuronal loss in the PFC. Similarly, no neuronal loss was seen across multiple rhesus macaque brain regions, including the PFC, hypothalamus, and visual cortex (Peters et al., 1994, 1998; Roberts et al., 2012; Giannaris and Rosene, 2012; Stonebarger et al., 2020). Notably, the hippocampus has not to our knowledge been stereologically analyzed across age in the rhesus macaque. This brain region is highly sensitive to cognitive and pathological aging changes, critical to memory consolidation, and undergoes significant shrinkage in AD (Freeman et al., 2008; Squire et al., 2015). While the lack of neuron loss in all other analyzed areas seems to indicate that rhesus macaques do not naturally lose neurons as they age, it is interesting to note that the multiple cohorts of rhesus monkeys have shown p-tau tangles in the hippocampus (i.e., Paspalas et al., 2018; Datta et al., 2021). Furthermore, in clinical studies it has been shown that p-tau tangles are associated with cell death—one of the primary drivers of cognitive decline in AD (Peters et al., 1994; Datta et al., 2021). Taken together, the overall consensus is that rhesus macaques do not show the distinctive neuronal loss that characterizes the later stages of AD in humans, and which is associated with dementia.

## Conclusion and Future Perspectives

**FIGURE 2:**
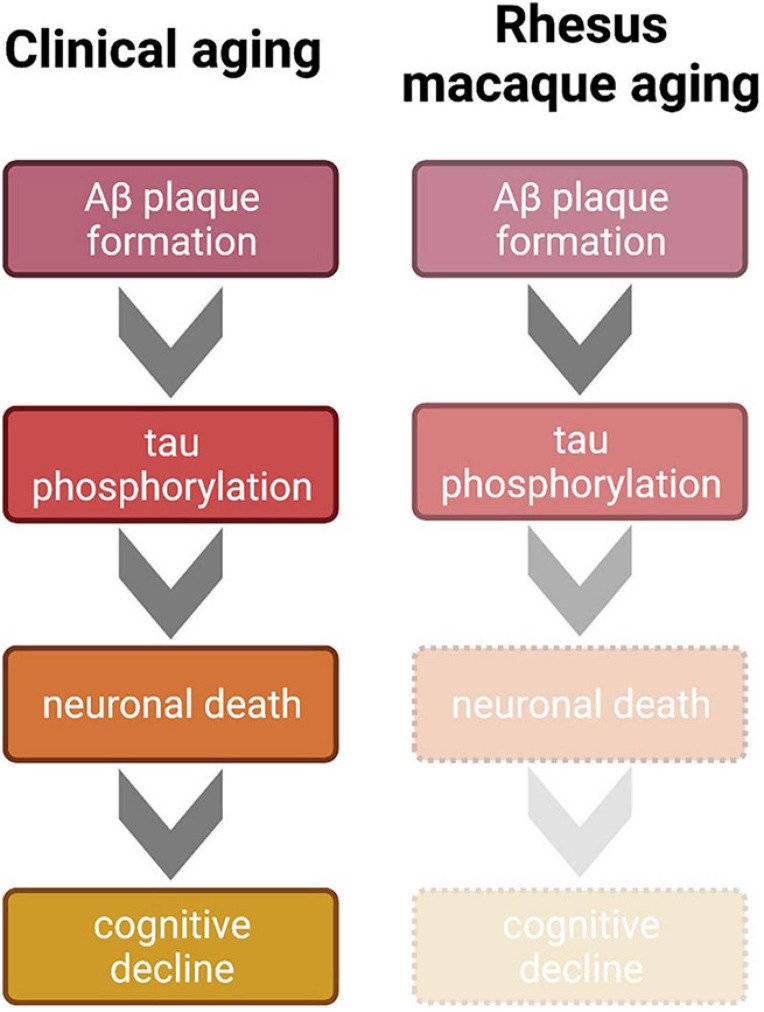
Comparison of clinical and non-human primate progression of brain aging according to the generally accepted amyloid cascade hypothesis of AD. Clinically, Aβ aggregates and triggers hyperphosphorylation of tau. This tauopathy accumulates intracellularly to lead to neuronal death, which is a primarily proposed mechanism of cognitive decline. Mid to late stages of AD result in the full clinical cascade visualized on the left. However, even in the oldest known rhesus macaques, this trajectory is incomplete; Aβ plaques and tauopathy do not reach levels seen in the clinical AD cases. Therefore, because tauopathy only reaches early stages of phosphorylation, cell death and the subsequent sharp cognitive decline are absent in the monkey model, which renders the rhesus macaque more suitable for naturally modeling normative clinical brain aging or early stages of AD. Figure created with BioRender.com.

Clinical studies can guide future directions of rodent behavioral and molecular research with regard to normal and pathological aging, which in turn can inform non-human primate research thereby potentially impacting clinical outcomes. This cross-species integrative approach is impractical, however, without detailed characterization of aging and AD-like symptomology in the most common preclinical non-human primate: the rhesus macaque. Importantly, in the rhesus macaque model of aging, all four major hallmarks of AD—significant cognitive decline, amyloid beta plaques, p-tau tangles, and neuronal death—do not reach the pathological levels of clinical AD. Cognitive decline in aged rhesus monkeys parallels the healthy aging human very well, in that gradual, incremental decline is seen across age, beginning in middle age or later, depending on the task. Amyloid beta plaques do naturally accumulate in the rhesus macaque brain, especially in sensitive areas such as the PFC. Importantly, however, the area coverage of these plaques in rhesus monkey brains is not high enough to be considered an AD phenotype relative to diagnostic hallmarks in the clinic. While the recent discovery of naturally occurring p-tau in the rhesus monkey brain is indeed exciting, data thus far indicate that the amount of p-tau detected is not in line with the classically accepted progression and advanced conditions of neurofibrillary tangles that represent hallmarks of AD. However, four distinct patterns of tau progression have recently been proposed; therefore, it is unclear which of these trajectories monkey p-tau most closely resembles (Vogel et al., 2021). Finally, neuronal death is an established AD phenotype, but this hallmark has not been obvious even in the very oldest rhesus macaques in studies to date. Taken altogether, it is evident that the rhesus macaque does not develop overt AD, even though the CNS conditions represent subclinical variations of AD hallmarks, as visualized in Figure 2. Notably, this environment can be experimentally leveraged in a systematic fashion, setting the stage for induction or mitigation models in which various methods can be employed to exacerbate or attenuate existing pathology (i.e., Beckman et al., 2021).

Due to these temporal restrictions along with the very recent discovery of naturally arising p-tau in the rhesus macaque, true non-human primate models of AD are severely lacking. Monkeys, like humans, show great variability in phenotypical aging, meaning even in aged animals these hallmarks are not present in the entire population. This makes a natural aging macaque an expensive and unreliable model for AD. This highlights the need for inducible non-human primate models of neurodegeneration. Notably, this environment has begun to be experimentally leveraged in a systematic fashion, setting the stage for induction or mitigation models in which various methods can be employed to exacerbate or attenuate existing pathology (i.e., Beckman et al., 2021). For instance, both rhesus and cynomologus macaques—a close genetic relative—have been shown to develop p-tau tangles in response to the injection of Aβ oligomers; however, even when induced tangles aggregate earlier in the lifespan, there is no recorded cell death (Lyra e Silva et al., 2019). Importantly, these are early experimental models, and work continues to be done to both characterize and exacerbate brain pathology in the aging monkey. Therefore, the rhesus macaque, while not an ideal natural model of clinical AD, has the potential to be an excellent resource with which to study factors that contribute to the etiology, exacerbation, and root causes of the discussed mechanisms, including genetics, hormone milieu, diet, trauma, and infection.

## Author Contributions

GS drafted the current manuscript, with writing and editing contributions from HB-N and HU. All authors contributed to the article and approved the submitted version.

## Conflict of Interest

The authors declare that the research was conducted in the absence of any commercial or financial relationships that could be construed as a potential conflict of interest.

## Publisher’s Note

All claims expressed in this article are solely those of the authors and do not necessarily represent those of their affiliated organizations, or those of the publisher, the editors and the reviewers. Any product that may be evaluated in this article, or claim that may be made by its manufacturer, is not guaranteed or endorsed by the publisher.
